# Epidemiology of spinal cord injuries in three selected counties in Kenya

**DOI:** 10.4102/sajp.v81i1.2097

**Published:** 2025-01-31

**Authors:** George M. Muli, Veronica Ntsiea, Natalie Benjamin-Damons, Nassib Tawa

**Affiliations:** 1 Department of Physiotherapy, Faculty of Therapeutic Sciences, University of the Witwatersrand, Johannesburg, South Africa; 2 Department of Rehabilitation Science, School of Medicine, Jomo Kenyatta University of Agriculture and Technology, Nairobi, Kenya

**Keywords:** complication, epidemiology, prevalence, spinal cord injury, risk factors and characteristics of SCI

## Abstract

**Background:**

Spinal cord injury (SCI) presents a significant health challenge, characterised by damage to the spinal cord resulting from trauma, inflammation, tumours or other aetiologies. This injury often leads to a range of debilitating consequences, including loss of motor function, sensation, sphincter control and autonomic nerve function below the site of injury, as well as challenges with self-care and performance of instrumental activities of daily living (ADLs).

**Objectives:**

This study aimed to determine the prevalence, risk factors and characteristics of SCI among adult patients in three selected counties in Kenya.

**Method:**

A population of 213 patients from three counties in Kenya was used for this study. Then, a retrospective descriptive cross-sectional study design was utilised to evaluate the prevalence of SCI.

**Results:**

The findings reveal a predominant prevalence in male patients, accounting for 84.04% of occurrences, with the highest incidence observed among individuals aged 26–35 years (36.15%). Motor vehicle accidents are the leading cause, accounting for 45.07% of cases, followed by falls from height accounting for 42.25% of cases and violence, specifically gunshot injuries, which account for 7.04% of incidents.

**Conclusion:**

The findings of this study provide a comprehensive epidemiology of SCI in three counties in Kenya with male patients recording high prevalence in motor vehicle accidents and falls from height as well as in severity and associated SCI complications.

**Clinical implications:**

This finding provides significant information on safety awareness and a platform to develop rehabilitation programmes for patients with SCI in Kenya.

## Introduction

The World Health Organization (WHO) estimates that globally, there are approximately 15.4 million people living with spinal cord injury (SCI) (Lu et al. 2024). Wyndaele and Wyndaele (2006) and Naidoo (2018) reported approximately 10.4–83 cases of SCI per million people worldwide annually. In high-income countries, the incidence of SCI varies from 13.1 (Ireland) to 163.4 (Canada) cases per million people per year, while in low-income countries, it ranges from 13.0 (Botswana) to 220.0 (Estonia) cases per million people per year (Kang et al. 2018). A comparative study conducted by Chiu et al. (2010) between low- and middle-income countries (LMICs) found that the incidence rates for traumatic SCI in middle-income countries ranged from 12.7 to 29.7 cases per million people per year, which is higher than that in high-income countries. Rahimi-Movaghar (2013) reported an annual incidence of 2.1 (Saudi Arabia) to 13.07 (Bulgaria) cases of SCI per million people in middle-income countries. Additionally, a study in Tanzania (a low-income country) by Moshi et al. (2017) reported an incidence of 26 cases of SCI per million people per year. Overall, SCI is a debilitating condition that causes severe and irreversible physical and psychological disabilities, significantly impacting the quality of life for patients (Kriz et al. 2017).

Spinal cord injury occurs when the spinal cord is damaged by trauma, inflammation, tumours or other causes, leading to a loss of motion, sensation, sphincter control and autonomic nerve function below the affected area (Yang et al. 2014). Based on the cause, SCI can be categorised into two groups: traumatic spinal cord injuries (TSCIs) and non-traumatic spinal cord injuries (NTSCIs). Traumatic spinal cord injuries are caused by external forces like motor vehicle accidents (MVAs), falls, violence or other factors (Moshi et al. 2017). Non-traumatic spinal cord injuries, on the contrary, involve underlying pathologies such as bleeding, infectious diseases, tumours, musculoskeletal diseases like osteoarthritis and congenital conditions (O‘Connor 2002, 2006). The severity of motor, sensory and autonomic neurological impairments resulting from SCI varies depending on the level and extent of the SCI (Walters et al. 2013; Wu et al. 2013).

The American Spinal Injury Association (ASIA) impairment scale classifies SCIs as complete and incomplete based on the absence or preservation of sensory and motor functions at the S4–S5 level. An SCI is considered complete if there is no sensory or motor function in that area. Incomplete SCIs refer to cases where some sensory or motor function is preserved below the level of injury, including the S4–S5 segments, which can still result in severe impairments (Scivolleto et al. 2015). The extent of neurological recovery depends on the degree of impairment, with most recovery occurring within the first 3 months after the injury. Complete lesions rarely experience full recovery, while incomplete lesions have a higher potential for recovery (Derakhshanrad et al. 2015).

Rehabilitation through physiotherapy plays a crucial role in achieving the primary goals of care for patients with SCI. Physiotherapy is essential in promoting motor recovery, enhancing posture control and improving mobility, which are keys to maximising independence and enabling participation in daily activities. Effective physiotherapy interventions are tailored to individual needs, focusing on strengthening the muscles that remain functional, improving balance and coordination, and retraining movement patterns to compensate for lost function.

Physiotherapy also contributes significantly to the psychological well-being of patients by helping them regain a sense of autonomy and self-efficacy. The process of rehabilitation empowers individuals to adapt to their new physical circumstances, fostering resilience and improving quality of life. Given the variability in health care systems and approaches to care between countries, the role of physiotherapy in SCI rehabilitation highlights the need for reliable epidemiological data (Guantai et al. 2019; Mbuthia 2016). Such data are essential for planning and delivering appropriate health services that are accessible and tailored to the needs of SCI patients, ensuring equitable allocation of resources (Brouwers et al. 2020; Sramkova et al. 2017). Therefore, this study aims to conduct an epidemiology of SCI in three selected counties (Nairobi, Machakos and Mombasa) in Kenya to establish the prevalence of SCI.

## Research methods and design

### Study design

This study utilised a retrospective descriptive cross-sectional study design to determine the prevalence, cause and characteristics of patients with SCI in three selected counties in Kenya. (Note that here, ‘retrospective’ means we collected and analysed pre-existing data from patient records rather than prospectively gathering new data, while the cross-sectional approach refers to snapshot retrieval of information from past records, hence the retrospective nature). Medical records of all patients diagnosed with SCI, regardless of the cause (traumatic or non-traumatic), were retrospectively reviewed for a 4-year period (2016–2020) at Cost General Hospital, Kenyatta National Hospital and Machakos Level 5 Hospital. This study included adults aged 18 years and older who had a confirmed diagnosis of SCI, while excluding patients with secondary conditions such as head injuries and unconfirmed SCI diagnoses.

### Research setting and participants

The study took place in Kenya, specifically in the counties of Nairobi, Machakos and Mombasa. These counties were selected as data collection areas because of their significant populations. Both Nairobi and Mombasa counties have all the hospital levels, but Machakos County has levels two to five hospitals. Here, level refers to the services offered based on specialisation. Additionally, Nairobi County has a specialised SCI hospital (National Spinal Cord Injury Hospital), the only one in the entire country.

The three hospitals were selected based on the levels (i.e. all serve as referral hospitals from other minor hospitals in the county) and services offered (i.e. in-patient and out-patient). In addition, the three counties were selected because of high cases of SCI.

The study focused on tertiary and level 5 hospitals within these counties. The total population of Kenya in 2019, according to the Central Bureau of Statistics (CBS), was 47 564 296. In the data collection counties, the populations were as follows: Nairobi – 4 397 073, Machakos – 1 421 932 and Mombasa – 1 208 333 (CBS 2019). The study aimed to identify and analyse patients with SCI in these counties. In 2020, the estimated number of SCI survivors in each county was approximately 264 in Nairobi, 85 in Machakos and 72 in Mombasa. This was obtained through WHO estimates of global incidence given as between 40 and 80 cases per million (WHO & ISCS 2013). The researchers accessed the records of individuals with SCI from the selected hospitals in these counties. To determine the sample size for the study, the Yamane formula (1992) was used. Based on this calculation, the estimated study population in the three counties was 455 individuals with SCI. In this study, sample size was determined using the Yamane formula (Equation 1):

$$n=\frac{N}{1+N{\left(e\right)}^{2}}$$ [Eqn 1]

Where *n* is the sample size, *N* is the population size and e is the level of precision (i.e. 0.05).

The sample sizes for each county were determined as follows: Nairobi – 159, Mombasa – 70 and Machakos – 61 persons with SCI. According to our calculation, the expected population sample for this study was 290 patients with SCI; however, based on the inclusion criteria, we were able to recruit 213 patients with SCI during the actual study. In addition, some hospitals like Coast General Hospital and Machakos Level 5 hospital lacked digital patient records, hence leading to inadequate patient data.

To be included in the study, participants, that is, the individuals whose medical records were reviewed, had to have a confirmed clinical diagnosis of SCI as per hospital records, be 18 years of age or older, and have been admitted to one of the selected hospitals.

### Inclusion and exclusion criteria

In this study, we excluded individuals with SCI who also had secondary conditions to maintain a focused and homogeneous study population. We aimed to isolate the effects of SCI on the outcomes of interest without the potential confounding influence of additional medical conditions, which could have introduced variability and made it more challenging to interpret the results. However, for this study, our primary objective was to establish a clear baseline understanding of SCI outcomes, which necessitated this exclusion.

### Data collection procedure

In this study, data were obtained from the hospital archives and wards in both tertiary and level 5 hospitals in the three selected counties.

After receiving approval, we accessed the patients’ records, which included information such as socio-demographic characteristics, the cause and date of SCI, whether the injury was complete or incomplete, complications and the level of the spinal cord lesion.

This information was collected through a data capture sheet as a standardised data collection form to extract specific information from hospital files, rather than a survey conducted through direct interaction with patients or staff. To make the data collection process more efficient, we hired a research assistant in each county to review and extract relevant patient record information. To ensure accuracy and consistency, the team followed standardised protocols and implemented several bias-reduction strategies, including blinding, randomisation and quality checks. In this study, individual was selected based on their extensive expertise. Specifically, they hold relevant degrees, certifications or titles and have at least 3 years’ relevant experience in the field which makes them particularly well-suited to provide insights and contribute to this study.

### Validity and reliability test

This study aimed to evaluate the validity and reliability of an instrument by conducting a series of tests. Firstly, content and construct validity were established by consulting experts who reviewed the instrument to ensure it accurately reflected the constructs being measured. Secondly, feedback was used to make necessary adjustments. Factor analysis confirmed that the instrument effectively captured the underlying dimensions of the constructs. For reliability, internal consistency was checked using Cronbach’s alpha, and test–retest reliability was assessed to ensure stability over time. The instrument was found to be both valid and reliable, with a Cronbach’s alpha of 0.90, indicating excellent internal consistency (Table 1). This aligns closely with previous studies that reported high reliability and good discrimination of similar instruments.

**TABLE 1:** Reliability tests.

Variable	Measures	Cronbach’s alpha	Number of items
Level of SCI	Cervical	0.896	12
	Upper thoracic	-	-
	Mid-thoracic	-	-
	Lower thoracic	-	-
	Lumbar	-	-

SCI, spinal cord injury.

### Sampling adequacy

The study utilised variables and tests of sampling adequacy to assess validity. Specifically, Bartlett’s test of sphericity was employed to determine the redundancy among variables, which could be effectively summarised with fewer factors (Williams et al. 2010; Yong & Pearce 2013). For factor analysis to be appropriate, the test should yield a significant result (*p* < 0.05). Table 2 shows Kaiser–Meyer–Olkin (KMO) test of sampling adequacy and Bartlett’s test of sphericity. According to the results, the scales for the level of SCI (0.854) exceeded the 0.7 threshold set by Williams et al. (2012), indicating strong reliability. The KMO measure of sampling adequacy was above the acceptable level of 0.5. Bartlett’s test of sphericity showed *p*-values below 0.05 (*p* < 0.001), confirming the data’s suitability for factor analysis. Additionally, determinant values for the level of SCI (0.085) were positive, further supporting the validity of the analysis.

**TABLE 2:** Kaiser–Meyer–Olkin and Bartlett’s test.

Factors (Domains)	KMO test	Bartlett’s test of sphericity			Determinant
		Approx. Chi-square	*df*	Sig.	
Level of SCI	0.854	312.897	8	0.006	0.085

KMO, Kaiser–Meyer–Olkin; SCI, spinal cord injury; *df*, degree of freedom; Sig., significance.

### Pilot testing

We also conducted a pilot study for the use of data capture sheets by the field data collectors to check their understanding on the use of the instrument. Feedback from this pilot study was then used to make the final data capture sheet to ensure that the instrument performed well in practice.

### Ethical considerations

Ethical clearance was obtained from the University of Nairobi Faculty of Health Sciences Ethics and Research Committee (reference no.: KNH-ERC/A/362). To ensure compliance with ethical guidelines, formal approval was obtained before accessing the patient’s files, from the Ministry of Health (MOH), National Commission for Science, Technology and Innovation (NACOSTI), Kenyatta National Hospital (KNH) and the respective county research committees. In addition, to protect participants’ privacy, we used codes rather than names, demographics, and injuries. Data analysis was carried out retrospectively using pseudonymised data. We recorded the patient data in Excel files and saved it on the computer. This was consistent with the Helsinki recommendations and the Ministry of Health, Republic of Kenya’s guidelines for human research participants.

### Data preparation and analysis

Our study data were initially recorded in Microsoft Excel spreadsheets, ensuring accuracy and consistency by organising and labelling each variable and data entry. The data were then exported to a compatible format for transfer to SPSS® version 25. The transfer process involved importing the data into SPSS using the ‘File’ menu and mapping the variables as defined in Excel. Finally, we verified and validated the imported data to ensure accuracy and maintain dataset integrity before conducting statistical analyses. Data analysis was conducted on the entire dataset, with additional comparisons made based on facility and age. The descriptive statistics are calculated and presented in summary tables. Chi-square test of association between socio-demographic characteristics was calculated. The prevalence of SCI in the three selected counties in Kenya was then estimated for the years 2016 to 2020 (Mbuthia 2016).

## Results

### Socio-demographic characteristics and cause of spinal cord injury

In this study, the expected population sample was 290 patients with SCI; however, based on the inclusion criteria and lack of digital data in Mombasa and Machakos counties, we were able to recruit 213 (159 + 44 + 10) patients as mentioned in the setting and participation section. According to Table 3, the results show insights into the socio-demographic characteristics of patients with SCI. When considering gender, most of the patients with SCI were male, accounting for 84.04% (*n* = 179), and 34% (*n* = 34) were female; in terms of age, the highest number (36.15%, *n* = 77) of patients admitted with SCI were within the age range of 26 to 35 years. The second-highest age group was between 36 and 45 years (21.60%, *n* = 46). The lowest number of people with spinal injury was in the age category of 75 years and above (0.94%, *n* = 2). Regarding levels of education, a significant proportion of SCI patients had a primary level of education, making up 50.23% (*n* = 107) of the cases. The next most common level of education was secondary education, representing 38.03% (*n* = 81) of the patients. Only two (0.94%) patients had a university level of education. In summary, the data revealed that many patients with SCI were male, primarily between the ages of 26 and 35 years, and had a primary level of education.

**TABLE 3:** Socio-demographic and cause of spinal cord injury.

Variables	*n*	%
**Gender**		
Male	179	84.04
Female	34	15.96
Total	213	100.00
**Age (years)**		
18–25	30	14.08
26–35	77	36.15
36–45	46	21.60
46–55	30	14.08
56–65	16	7.51
66–75	12	5.63
75 and above	2	0.94
Total	213	100.00
**Level of education**		
Primary	107	50.23
Secondary	81	38.03
College	23	10.80
University	2	0.94
Total	213	100.00
**Causes of SCI**		
Accident during sports	3	1.41
Disease, for example, infections	9	4.23
Fall from height	90	42.25
Violence, for example, gunshot	15	7.04
Motor traffic accident	96	45.07
Total	213	100.00

SCI, spinal cord injury.

The most prevalent cause was motor traffic accidents, accounting for 45.07% (*n* = 96) of the cases. The second most common cause was falls from height, comprising 42.25% (*n* = 90) of the cases. There were a few instances of SCI resulting from sports-related accidents, which constituted 1.41% (*n* = 3) of the total cases. Additionally, a small proportion of cases, 4.23% (*n* = 9), were attributed to disease. However, 7.04% (*n* = 15) of the reported cases were as a result of gunshot injuries.

### Lesion level and complications

The results presented in Table 4 illustrate the distribution of the level of SCI according to gender and the complications following SCI. The most common type of SCI observed in both male patients and female patients was cervical lesions, accounting for 46.01% of cases. This was followed by lumbar lesions at 19.72% and lower thoracic lesions at 18.78%. The incidence of thoracolumbar lesions was the lowest (0.94%). When considering gender-specific data, it was found that cervical lesions were more prevalent among male patients, comprising 39.44% of cases (*n* = 84). Lower thoracic and lumbar lesions were the next most common among male patients, accounting for 16.90% (*n* = 36) and 15.96% (*n* = 34), respectively. Cervical lesions were the most frequent type of SCI, representing 6.57% (*n* = 14) of cases, followed by lumbar lesions at 3.76% (*n* = 8) in female participants.

**TABLE 4:** Level of spinal cord injury by gender distribution and complications.

Variables	Female		Male		Total	
	*n*	%	*n*	%	*n*	%
**Level of SCI**						
Cervical	14	6.57	84	39.44	98	46.01
Upper thoracic	2	0.94	16	7.51	18	8.45
Mid-thoracic	6	2.82	7	3.29	13	6.10
Lower thoracic	4	1.88	36	16.90	40	18.78
Thoracolumbar	0	0.00	2	0.94	2	0.94
Lumbar	8	3.76	34	15.96	42	19.72
Total	34	15.96	179	84.04	213	100.00
**SCI complications**						
Pressure ulcers	1	0.47	7	3.29	8	3.76
Urinary tract infection	0	0.00	9	4.23	9	4.23
Autonomic dysreflexia	9	4.23	38	17.84	47	22.06
Spasticity	7	3.29	23	10.80	30	14.08
Neuropathic pain	15	7.04	75	35.21	90	42.25
Pulmonary complications	2	0.94	2	0.94	6	2.82
Venous thromboembolism	0	0.00	1	0.47	1	0.47
Astasis	0	0.00	17	7.98	17	7.98
Others	0	0.00	7	3.29	5	3.29
Total	34	15.96	179	84.04	213	100.00

SCI, spinal cord injury.

This study also investigated the complications associated with SCI and their distribution across genders. The most common complication reported in both male patients and female patients was neuropathic pain, affecting 42.25% of cases (*n* = 90). This was followed by autonomic dysreflexia at 22.06% (*n* = 47) and spasticity at 14.08% (*n* = 30). The occurrence of venous thromboembolism was the least frequent complication, observed in only 0.47% of cases (*n* = 1). Overall, the results indicate that male patients experienced a higher incidence of complications compared to their female counterparts. Findings are summarised in Table 4, providing valuable insights into the nature and prevalence of spinal cord injuries in Kenya.

### Types of spinal cord injury

There were a large number of incomplete lesions in the cervical segment (37.09%, *n* = 79) followed by lumbar lesions (16.43%, *n* = 35) and lower thoracic lesions at 13.15% (*n* = 28). Overall, incomplete spinal lesions accounted for 76.53% (*n* = 163) of all the SCI levels.

The study also examined the relationship between the severity of the injuries and their causes. To determine the severity of injuries, radiological tools (CT scan and MRI) and clinical examination records (motor, sensory, reflexes and autonomic functions) were used.

As shown in Table 5, it was observed that most patients with incomplete lesions had suffered traffic accidents, accounting for 45.07% (*n* = 96) of the cases. Falls from a height were the second most common cause, with 42.25% (*n* = 90) of the patients experiencing incomplete lesions. Sports-related accidents had very low occurrences of both complete and incomplete cases, with only 0.94% (*n* = 2) and 0.47% (*n* = 1), respectively. Additionally, Table 5 provides a summary of the causes of SCI in relation to the extent of the lesion at the time of injury. From the results presented in Table 5, it is evident that neuropathic pain was the most prevalent complication experienced by SCI patients, accounting for 40.38% (*n* = 86) of the cases. Autonomic dysreflexia was the second most common complication, affecting 26.76% (*n* = 57) of the patients. Neuropathic pain was found to be more commonly associated with complete lesions. Lastly, venous thromboembolism was reported in very few cases of both complete and incomplete severity among SCI patients.

**TABLE 5:** The level of spinal cord injury, cause of injury and complications in relation to the severity of spinal cord injury.

Variables	Complete		Incomplete		Total	
	*n*	%	*n*	%	*n*	%
**Level of SCI**						
Cervical	19	8.92	79	37.09	98	46.01
Lower thoracic	12	5.63	28	13.15	40	18.78
Mid-thoracic	4	1.88	9	4.23	13	6.10
Upper thoracic	6	2.82	12	5.63	18	8.45
Thoracolumbar	2	0.94	0	0.00	2	0.94
Lumbar	7	3.29	35	16.43	42	19.72
Subtotal	50	23.47	163	76.53	213	100.00
**Cause of injury**						
Accident during sport	2	0.94	1	0.47	3	1.41
Disease, for example, infection	6	2.82	3	1.41	9	4.23
Fall from height	21	9.86	71	33.33	90	42.25
Violence, for example, gunshot	9	4.23	6	2.82	15	7.04
Traffic accident	12	5.63	82	38.50	96	45.07
Subtotal	50	23.47	163	76.53	213	100.00
**SCI complications**						
Pressure ulcers	1	0.47	5	2.35	6	2.82
Urinary tract infection	1	0.47	5	2.35	6	2.82
Autonomic dysreflexia	16	7.51	41	19.25	57	26.76
Spasticity	7	3.29	24	11.27	31	14.55
Neuropathic pain	17	7.98	69	32.39	86	40.38
Pulmonary complication	4	1.88	4	1.88	8	3.76
Venous thromboembolism	0	0.00	1	0.47	1	0.47
Astasis	4	1.88	9	4.23	13	6.10
Others	0	0.00	5	2.35	5	2.35
Subtotal	50	23.47	163	76.53	213	100.00

SCI, spinal cord injury.

## Discussion

Spinal cord injuries represent a significant public health challenge globally, with considerable implications for health care systems, particularly in LMICs like Kenya. These injuries often lead to profound physical, psychological and socio-economic consequences for individuals, further complicating the delivery of effective health care. In Kenya, where digital health record systems are not uniformly implemented, understanding the epidemiology of SCI is crucial for improving patient outcomes and informing policy decisions.

The findings of this study provide important insights into the epidemiology of SCI and align with existing research, reinforcing certain patterns while also highlighting areas that require further attention. The observation that MVAs and falls from height are the leading causes of SCI is consistent with existing literature. For instance, Khazaeipour et al. (2014) and Kang et al. (2018) also identified MVAs as a predominant cause of SCI, emphasising the need for targeted preventive measures. These results underscore the urgency of implementing safety campaigns, enhancing road safety regulations and designing fall prevention programmes, particularly in high-risk environments such as construction sites.

In contrast, our study has reported relatively low cases of sports-related injuries as a major cause of SCI unlike suggested in other studies (Bureau of Spinal Cord Injury Research 2016). This contradiction indicates that while sports-related SCIs may be less frequent, they still represent a critical cause of SCI which requires preventive intervention, particularly in high-contact sports.

The study also indicates the significant, yet often under-discussed, issue of gunshot-related spinal injuries. The presence of such injuries points to a broader societal problem, namely, violence, which is increasingly recognised as a public health issue. As noted by Rahman et al. (2018), communities experiencing higher rates of violence also see a corresponding rise in trauma-related SCIs. This correlation suggests that addressing the root causes of violence through community engagement, law enforcement and policy initiatives could play a crucial role in reducing gunshot-related SCIs.

Moreover, the demographic patterns observed in this study, particularly the gender disparity in SCI rates, align with previous research, which consistently shows a higher incidence of SCIs among male patients. The study by Rahman et al. (2018) in Bangladesh reported a similar male predominance, which can be attributed to higher engagement in risk-taking behaviours among men. This finding has significant implications for preventive strategies, suggesting that interventions should not only focus on high-risk activities but also consider gender-specific approaches to reduce SCI incidence. Generally, this study reaffirms the importance of focusing on preventive measures for the most common causes of SCI – MVAs and falls – while also addressing less common but critical causes such as sports injuries and gunshot wounds (Zhang et al. 2021).

The data from our study revealed that a significant proportion of SCI occurred among young individuals aged 45 years and below, with a notable 50.23% of these individuals having lower levels of education. These findings are consistent with previous research, such as Khazaeipour, Taheri-Otaghsara and Naghdi (2015); Betthauser et al. (2022); and Guantai et al. (2021), which show that lower education and income levels are associated with an increased risk of SCI. This correlation suggests that young adults with limited educational attainment are often relegated to low-paying, high-risk jobs. Their educational background, often insufficient for securing less physically demanding employment, likely contributes to their vulnerability to SCI, especially those resulting from traumatic incidents, a trend also observed by Wang et al. (2013).

Moreover, the intersection of poverty and disability, as explored by Braithwaite and Mont (2009), further supports the notion that socio-economic disadvantages can exacerbate the risk of SCI. The confluence of low income, limited educational opportunities and employment in high-risk occupations creates a cycle of vulnerability for young adults, particularly in physically demanding sectors. This situation underscores the need for targeted interventions in education, vocational training and public health policy.

In line with this, it is crucial to emphasise the role of education in safety awareness, particularly in preventing falls from heights, a common cause of SCI in occupations such as construction and agriculture. Sharwood et al. (2018) reported that a significant number of SCIs among casual workers in these sectors are because of falls from heights. Additionally, sporting activities, a prevalent cause of SCI among youth, as noted by Kim et al. (2011) and Ding et al. (2022), further illustrate the diverse contexts in which young adults are at risk.

These findings call for a comprehensive approach to SCI prevention that includes enhancing educational opportunities, improving safety standards in high-risk occupations and promoting awareness about the risks associated with both work-related and recreational activities. Addressing these factors could reduce the incidence of SCI among vulnerable populations and improve overall health and economic outcomes.

Our study also reported MVAs (45.07%) and falls from height (42.25%) as the predominant causes of SCI across three counties, aligning closely with studies by Kamardeen (2022) and Wakhayanga (2022) who reported a high prevalence of SCI resulting from falls from ladders, particularly among casual workers, while Barbiellini (2022) and Mehdar et al. (2019) reported MVAs as a significant cause of SCI. These findings affirmed the notion that certain occupational hazards and high-risk activities, such as driving and working at heights, are primary contributors to SCI, necessitating targeted preventive measures.

Regarding the anatomical distribution of SCI, cervical injuries were the most common (46.01%), followed by lumbar injuries (19.72%). This pattern is consistent with the results of Yang et al. (2014), who reported a higher incidence of cervical injuries (1720 cases) compared to thoracic injuries (1264 cases) in a Chinese cohort. Ning et al. (2011) similarly observed that cervical lesions were the most frequent, with thoracic lesions ranking third. The vulnerability of the cervical spine, as noted by Sekhon and Fehling (2001), can be attributed to its high mobility, its role in balancing the cranium and the relatively small size and reduced strength of the vertebrae, ligaments and muscles in this region. The connection of the cervical spine to the more rigid and stable thoracic cage may also contribute to its susceptibility to injury. Conversely, the thoracic region’s stability, provided by the rib cage and stronger vertebrae, generally offers greater protection, making it less prone to injury.

Moreover, our findings also suggest that the higher cases of quadriplegia may be linked to improved clinical management and handling of patients at the accident scene, which enhances the likelihood of survival following high cervical spinal injuries (Bromley 2006).

When examining complications associated with SCI, our study identified neuropathic pain (42.25%), autonomic dysreflexia (22.06%) and spasticity (14.08%) as the most prevalent, with a higher occurrence in men. These findings are consistent with previous studies, such as those by Dada and Ogunleye (2022); Pilusa, Myezwa and Potterton (2021); and Lofvenmark et al. (2016), which also reported pain and bladder problems as common complications of SCI. The occurrence of autonomic dysreflexia, particularly in patients with lesions at or above T6, aligns with the observations of Balik and Šulla (2022), who noted that this complication is most prevalent in individuals with injuries at or above this spinal level.

Finally, our study found that the majority of SCI cases involved incomplete lesions, with cervical lesions being the most common (37.09%), followed by lumbar (16.90%), lower thoracic (13.15%) and upper thoracic (5.63%) lesions. These findings are in line with the work of Wyndaele and Wyndaele (2006) and Yang et al. (2014), who reported a higher prevalence of incomplete over complete spinal cord lesions. The consistency of these results with other studies suggests that the mechanisms leading to incomplete lesions are more common or perhaps better managed, resulting in less severe damage to the spinal cord.

In addition, when we compared our findings with other research on the aetiology of SCI (Barbiellini et al. 2022; Golestani et al. 2022; Mehdar et al. 2019; Ning et al. 2012), we noted that the MVAs falls from height, gunshot injuries and sports remain significant risk factors for SCI across different populations (Rahimi-Movghar et al. 2013). However, when we consider other factors, socio-demographics including age, gender and socio-economic factors may also influence the cause of SCI. For instance, certain age groups may be more susceptible to specific types of SCI because of differing activity levels or vulnerabilities (Yoon et al. 2015). Moreover, gender differences in injury patterns and outcomes have also been observed, with male participants often at higher risk because of more frequent engagement in high-risk activities (Aminian et al. 2016). Regional differences in health care access, infrastructure and environmental hazards can affect SCI causes and recovery. In areas with limited access to emergency medical services or rehabilitation facilities, outcomes for SCI patients may be less favourable (Kim et al. 2018).

## Conclusion

In conclusion, this study provides comprehensive insights into the epidemiology of SCI in three selected counties in Kenya. The findings reveal several important patterns and trends. Firstly, most SCI cases occurred among male patients, particularly within the age range of 26 to 35 years. Additionally, a significant proportion of patients with SCI had a primary level of education. Secondly, motor traffic accidents and falls from height were identified as the primary causes of SCI, underscoring the need for preventive measures and targeted awareness campaigns. Cervical lesions were the most common type of SCI, followed by lumbar and lower thoracic lesions. Male patients experienced a higher incidence of complications, with neuropathic pain being the most prevalent. In terms of severity, incomplete injuries were more common in road traffic accidents, and falls from height were the main causes. These findings emphasise the importance of addressing the specific causes of SCI and implementing preventive strategies to reduce the incidence and severity of these injuries. Further research and interventions are needed to address the gender disparities observed and to enhance education and awareness about SCI in Kenya.
